# Effectiveness of a preoperative explanatory video system on patient acceptance and anesthesiologists’ workload: a questionnaire survey

**DOI:** 10.1186/s40981-025-00805-9

**Published:** 2025-07-24

**Authors:** Kohei Akemoto, Kotoe Kamata, Yu Kaiho, Eiko Onishi, Shizuha Yabuki, Takuya Shiga, Masanori Yamauchi

**Affiliations:** 1 Department of Anesthesiology and Perioperative Medicine, Tohoku University Graduate School of Medicine, 2-1 Seiryo-machi, Aoba-ku Sendai-shi, Japan; 2 Department of Intensive Care of Medicine, Tohoku University Graduate School of Medicine, 2-1 Seiryo-machi, Aoba-ku Sendai-shi, Miyagi 980-8575 Japan; 3 Experience Design and Alliance Section: EDAS, Tohoku University Hospital. 1-1 Seiryo-machi, Aoba-ku Sendai-shi, Miyagi 980-8574 Japan

**Keywords:** Patient comprehension, Preoperative education, Video-assisted learning, Anesthesiologist workload, Video-enhanced patient education

## Abstract

**Background:**

High-quality preoperative patient education is crucial for enhancing comprehension and reducing anxiety. However, anesthesiologists often face time constraints that limit the depth of preoperative consultations. To address this challenge, we implemented a tablet-based preanesthetic explanatory video preview system, allowing patients to view tailored video clips prior to their consultation. This study aimed to evaluate the system’s impact on patient acceptance and the workload of anesthesiologists.

**Methods:**

A questionnaire-based survey was conducted at our outpatient clinic between October 27 and November 20, 2023. A total of 121 patients and 20 anesthesiologists participated. Patients completed a three-point scale questionnaire assessing the video’s clarity, relevance, and overall satisfaction. Anesthesiologists provided feedback on the efficiency of consultations and workload.

**Results:**

Ninety-five percent of patients found the video “easy to understand,” and 96% expressed overall satisfaction. Thirteen out of 14 anesthesiologists reported that the video improved patient understanding and reduced consultation time, with an average time savings of 3.9 ± 3.2 min per patient. None reported an increase in workload.

**Conclusions:**

The preanesthetic video preview system improved patient comprehension and satisfaction while enhancing consultation efficiency. Standardizing preoperative education through video may optimize clinical workflow and reduce the burden on anesthesiologists. Further research is warranted to assess its applicability across diverse patient populations and surgical settings.

## Background

High-quality patient education is essential to ensure that patients are well-prepared and fully informed before surgery. The preoperative period is often characterized by heightened psychological distress and increased cognitive burden [1]. In recent years, technological advances have not only improved surgical and anesthetic care but also underscored the importance of personalized patient guidance. Patients should receive both general information about anesthesia and tailored advice that addresses their medical conditions and specific concerns.

Traditionally, patient education has been delivered through face-to-face interactions, supplemented by written materials such as educational leaflets [2]. However, written materials require active patient engagement [3], and studies have shown that video-assisted education results in higher satisfaction and better understanding of anesthesia procedures compared to verbal explanations or brochures alone [4–7]. Recent advances in technology have further facilitated the use of video in patient education, as tablets and other more accessible devices become increasingly available.

At the same time, the global shortage of anesthesiologists has led to increased workloads, particularly in Japan, where physicians face long hours and rigorous training demands [8]. In April 2024, Japan implemented the “Work-Style Reform for Physicians” to establish legal limits on working hours, thereby prompting the need for structural changes in clinical practice [9]. Given that anesthesia time is primarily dictated by operating room schedules, optimizing efficiency remains a challenge. Strategies such as task-sharing and task-shifting, which delegate responsibilities to other healthcare professionals, have been proposed to improve workforce utilization while maintaining patient safety. Although video-based patient education is not a new concept, the practical use of tablet-based systems in modern clinical settings remains underreported. This is especially pertinent in Japan, where anesthesiologists often contend with high patient volumes and extended working hours, underscoring the need for innovative approaches that support both quality of care and sustainable workloads.

To address both patient education needs and anesthesiologist workload, our department formed a workstyle reform working group in April 2022. At Tohoku University Hospital, a tertiary academic center with 1160 beds and 21 operating rooms, preanesthetic evaluations for elective surgical patients, especially those undergoing complex or high-risk procedures, are conducted by certified anesthesiologists. Each consultant typically evaluates approximately 15 patients per day, with each session lasting around 30 min. As part of the initiative, we developed and implemented a tablet-based explanatory video system in December 2022, designed to streamline preanesthetic education. The videos, filmed in our operating rooms and featuring our staff, deliver anesthesia-specific information tailored to each patient’s condition. This system was not intended to replace physician–patient consultations, but rather to enhance their efficiency and depth by reducing repetitive explanations and improving baseline comprehension.

One year after implementation, we conducted a questionnaire survey to assess the feasibility and impact of the system. This study aims to evaluate whether video-assisted education can simultaneously improve patient understanding and reduce the consultation burden on anesthesiologists.

## Methods

Between October 27 and November 20, 2023, we conducted a survey on video-assisted patient education using a three‐point scale questionnaire at the preanesthetic outpatient clinic of Tohoku University Hospital. In accordance with hospital regulations, the survey was exempt from Institutional Review Board approval, as it did not involve identifiable personal data. Furthermore, since the study consisted solely of noninvasive questionnaire-based assessments, it was not registered in a clinical trial registry. Patients (or their guardians) and anesthesiologists who provided written informed consent were included in the study.

Before undergoing a face-to-face preanesthetic consultation, all participating patients viewed video clips on a hospital-owned tablet. These videos were tailored to their specific anesthetic needs, with an overview of the five available topics provided in Appendix A. Immediately afterward, participants completed a questionnaire to evaluate the clarity, relevance, and acceptability of the video-based education (see Appendix B). Prior to completing the questionnaire, the study protocol was explained, and written informed consent was obtained. Because consent was obtained only after the video session, patients were unlikely to have been influenced by awareness of the upcoming evaluation during viewing. Following this process, patients received a standard preanesthetic interview and risk assessment conducted by a board-certified anesthesiologist.

During the interview, anesthesiologists confirmed details of the planned surgery, reviewed the patients’ medical history and current medications, assessed anesthesia-related risk factors, and conducted a question-and-answer session. Notably, anesthetic techniques and common complications were not reiterated, as these topics had already been addressed in the video clips.

To objectively assess improvements in patient understanding resulting from the video previewing system, anesthesiologists answered the same questions presented to patients in Appendix B. Additionally, to evaluate the video’s impact on reducing anesthesiologists’ workload, they responded to a separate set of questions outlined in Appendix C.

## Results

During the survey period, 121 outpatients and 20 anesthesiologists participated, yielding 100 patient responses and 14 anesthesiologist responses. The patient cohort consisted of 32 males and 68 females, ranging in age from 7 to 90 years, with a median age of 56 years. The number of patients who reviewed each video clip was as follows: 80 for general anesthesia, 22 for epidural anesthesia, 1 for spinal anesthesia, 1 for peripheral nerve block, and 13 for Cesarean section. The participating anesthesiologists had between 9 and 23 years of specialized experience.

Regarding comprehension, 95 patients rated the video content as “easy to understand.” Three reported difficulty understanding all the details, and two did not respond. Notably, the two patients who found “some parts of the video difficult to understand” required additional explanations for both general and epidural anesthesia.

Overall, patient satisfaction with the video previewing system was high at 96%, and 12 out of 14 anesthesiologists recognized its effectiveness (Table 1). Additionally, 13 anesthesiologists reported that the videos improved patient understanding and helped reduce their workload (Table 2). On average, they noted a reduction in consultation time of 3.9 ± 3.2 min per patient compared to the period before the system’s introduction.

**Table 1:** Patient and anesthesiologist questionnaire on the acceptance of the video preview system

		Patients (n = 100)	Anesthesiologists (n = 14)
System operation	Agree	27	0
Could you/your patients operate the tablet independently?	Neither	63	11
	Disagree	10	3
Content relevance	Agree	95	13
Was the video content easy to understand for you/your patients?	Neither	3	1
	Disagree	0	0
Viewing duration	Agree	3	0
Was the video viewing time too long for you/your patients?	Neither	96	11
	Disagree	1	3
Timing of video clip access	Agree	87	5
Was the timing of the video preview appropriate for you/your patients?	Neither	0	9
	Disagree	12	0
Satisfaction	Agree	96	12
Were you satisfied with the video?	Neither	3	2
	Disagree	0	0

**Table 2:** Anesthesiologist questionnaire on the impact of the video preview system on outpatient workload

		Anesthesiologists (*n* = 14)
Workload reduction	Agree	13
Has your workload been reduced?	Neither	1
	Disagree	0
Improving patient understanding	Agree	9
Do you feel that patient understanding has improved?	Neither	4
	Disagree	0

## Discussion

This questionnaire survey demonstrated that our preanesthetic explanatory video preview system was highly satisfactory among outpatients. Additionally, it appears to reduce the workload associated with preanesthetic consultations effectively.

As hospital admission-to-surgery intervals continue to shorten, optimizing patient preparation has become increasingly important. In our study, none of the anesthesiologists reported an increased workload because of the video system. Although consultation times were not quantitatively measured, physicians perceived a reduction of approximately 4 min per consultation compared to the pre-video period. As previous studies have shown, shortening interview duration while maintaining quality can contribute to significant cost savings [10].

For patients with cognitive decline, such as those with dementia, the shared viewing of the video by both patients and their family members provided a common reference point that facilitated understanding. While most patients, particularly older adults or those with cognitive decline, required assistance with tablet operation, this support was provided by outpatient clerical staff. Although this introduced a minor initial burden, the overall reduction in anesthesiologists’ consultation time effectively compensated for it. Once the staff became familiar with the process, the additional workload became negligible. Since non-physician staff can assist patients in viewing educational videos, anesthesiologists can focus more on addressing individual concerns and obtaining informed consent. Given its accessibility, cost-effectiveness, and ease of integration into clinical workflows, video-based education shows strong potential for enhancing preoperative patient education.

Based on the findings of this survey, our department has revised its preoperative education process. The video system has been expanded to include all surgical patients. A newly hired physician assistant now meets with inpatients who did not attend an outpatient consultation, ensuring they access the video content via QR code before the anesthesiologist’s preoperative visit. This additional video session, combined with reinforcement during the anesthesiologist’s interview using written materials, is expected to improve patient adherence to perioperative instructions. It is also anticipated to reduce the duration of anesthesiologists’ preoperative visits.

Video is widely recognized as an effective tool for patient education, as supported by a growing body of literature [11]. Ong et al. reported that video provides greater flexibility and autonomy in learning, allowing patients to view content at their own pace [1]. Video-based education provides standardized information, helping reduce disparities in patient knowledge that may result from verbal explanations alone. Physicians widely acknowledge that multimedia tools enhance patient comprehension of complex medical topics [12]. In our survey, most patients reported high satisfaction and improved understanding of the tablet-based video content (Table 1).

Our custom-made videos helped patients understand clinical procedures, potential complications, and the overall operating room environment. However, some handwritten comments revealed areas for improvement. Two patients who selected “neither” for content relevance (Table 1) expressed a desire for further explanation of the rationale behind their anesthesia method, notably the addition of regional anesthesia (e.g., epidural) to general anesthesia. This highlights an ongoing challenge: conveying the reasoning behind anesthetic planning. Such decisions are multifactorial, based on surgical invasiveness, patient comorbidities, and anticipated postoperative care needs. These factors should be more explicitly addressed when incorporating video-based education into preanesthetic consultations.

Despite these promising findings, our study has several limitations. First, patient background information was limited to age; other potentially influential factors, such as surgical invasiveness or prior experiences with anesthesia and surgery, were not collected. Second, selection bias may be present, as 17% of patients and 30% of anesthesiologists did not respond to the questionnaire. Third, patient understanding of anesthesia was assessed only subjectively; an objective evaluation would require specific questions addressing anesthetic concepts. Additionally, because the survey was conducted immediately after video viewing, responses may reflect initial impressions of clarity and accessibility rather than deep understanding or long-term retention. Finally, interview durations were not measured quantitatively, limiting our ability to directly compare consultation times before and after the video system’s implementation.

## Conclusions

Our questionnaire survey demonstrated that the implementation of a preanesthetic explanatory video preview system was well received by outpatients and contributed to a reduction in anesthesiologists’ workload. These findings highlight the potential of structured, video-based education to improve clinical efficiency and promote patient-centered care in the preoperative setting.

## Presentations

Parts of this report were previously presented as a poster at Euroanaesthesia 2024 in Munich, Germany, and the 71 st Annual Meeting of the Japanese Society of Anesthesiologists in Kobe, Japan.

## Data Availability

The datasets used and analyzed in this study are available from the corresponding author upon request.

## Appendix 1

### Video Content

The procedures are depicted using real video sequences from the operating room and animations illustrating the actual procedure. The explanations in all videos avoid complex medical terminology to ensure accessibility for patients without medical training. However, for clarity, appropriate medical terms are used in the descriptions below.

## General Anesthesia (Duration 771 sec)

### Procedure

Preoperative instructions on fasting guidelines and smoking cessation; attachment of an electrocardiogram, noninvasive arterial blood pressure monitoring, and pulse oximetry; anesthesia induction via peripheral vein; mask ventilation; intubation.

### Risks

Aspiration pneumonia, persistent hoarseness, dental damage, malignant hyperthermia, allergic reactions, anaphylactic shock, pulmonary thromboembolism, postoperative nausea and vomiting, postoperative delirium, hematoma and nerve damage due to intravenous catheter placement, urethral damage from urethral catheterization, positioning-related injuries (nerve compression, skin damage), and severe systemic complications (e.g., cardiac and pulmonary issues).

## Epidural Anesthesia (Duration 226 sec)

### Procedure

Anatomy of the puncture site; puncture; catheter placement.

### Risks

Postdural puncture headache, persistent nerve damage, epidural hematoma, epidural abscess, paraplegia.

## Spinal Anesthesia (Duration 409 sec)

### Procedure

Anatomy of the puncture site; puncture; injection.

### Risks

Postdural puncture headache, persistent nerve damage, epidural hematoma, epidural abscess, paraplegia, bladder dysfunction.

## Peripheral Nerve Block (Duration 266 sec)

### Procedure

Anatomy of the puncture site; administration of local anesthetic under ultrasound guidance; catheter placement.

### Risks

Persistent nerve damage, local anesthetic systemic toxicity, local abscess, hematoma, respiratory failure, hypotonia.

## Cesarean Section (Duration 470 sec)

### Procedure and Risks

This video summarizes the procedures and associated risks described above for general anesthesia, epidural anesthesia, spinal anesthesia, and peripheral nerve block, as applicable to cesarean section cases.

## Appendix 2

### Patient and Anesthesiologist Questionnaire on the Acceptance of the Video Preview System (English Version)

Please select one option for each question.

1. (System operation) Could you and your patients operate the tablet independently?
  1. Agree
  2. Neither
  3. Disagree
2. (Content relevance) Was the video content easy to understand for you/your patients?
  1. Agree
  2. Neither
  3. Disagree
3. (Viewing duration) Was the video viewing time too long for you/your patients?
  1. Agree
  2. Neither
  3. Disagree
4. (Timing of video clip access) Was the timing of the video preview appropriate for you/your patients?
  1. Agree
  2. Neither
  3. Disagree
5. (Satisfaction) Were you satisfied with the video?
  1. Agree
  2. Neither
  3. Disagree

## Appendix 3

### Anesthesiologist Questionnaire on the Impact of the Video Preview System on Outpatient Workload (English Version)

Please select one option for each question.

1. Has your workload been reduced?
  1. Agree
  2. Neither
  3. Disagree
2. Do you feel that patient understanding has improved?
  1. Agree
  2. Neither
  3. Disagree
